# Effect of 10% Carbamide Peroxide on Tooth Shade, Plaque Index and Gingival Index during Invisalign Treatment

**DOI:** 10.3390/dj9050048

**Published:** 2021-04-26

**Authors:** Dalia Seleem, Shaahin Dadjoo, Ambrose Ha, Caitlyn Santos, Sahar Mirfarsi, Karen Matsumura-Lem, David Lazarchik

**Affiliations:** 1 College of Dental Medicine, Western University of Health Sciences, 309 E. Second Street, Pomona, CA 91766-1854, USA; sdadjoo@westernu.edu (S.D.); ambrose.ha@westernu.edu (A.H.); caitlyn.santos@westernu.edu (C.S.); Sahar.Mirfarsi@westernu.edu (S.M.); kmatsumuralem@westernu.edu (K.M.-L.); dlazarchik@westernu.edu (D.L.); 2 Eastman Institute for Oral Health, Orthodontic Residency Program, University of Rochester, Rochester, NY 14620, USA

**Keywords:** gingival index, plaque index, carbamide peroxide, Invisalign

## Abstract

Invisalign has improved periodontal health in comparison to traditional metal wire braces. Due to a growing interest in attaining better teeth esthetics, there are more adults seeking Invisalign treatment. Ten percent carbamide peroxide (CP) breaks down to 6.5% urea and 3.5% hydrogen peroxide, which elevates oral pH, removes stains, and diminishes caries by inhibiting plaque formation. The aim of this study is to investigate whether 10% CP use during Invisalign treatment can enhance tooth shade esthetics while decreasing plaque levels and improving gingival health indices. Twenty-eight patients at Western University dental center undergoing Invisalign were assigned to two groups where the experimental group applied daily bleaching material (10% CP, Ultradent Inc., South Jordan, UT, USA), while the control group did not for 4 weeks. Tooth shade, plaque index (PI), and gingival index (GI) were assessed at baseline and in 2-week intervals for 6 weeks. Results showed that 10% CP had significant change in tooth shade over the 2- and 4-weeks periods (*p* < 0.05) and significantly reduced plaque and gingival indices (*p* < 0.05), with minimal to no relapse after 2 weeks post-op. Thus, applying CP at 10% may be a useful application during Invisalign treatment in improving teeth shade and overall gingival health.

## 1. Introduction

Invisalign system with clear thermoplastic polyurethane aligners was first introduced to orthodontic practices in 1996 as an alternative to metal wire orthodontics [1,2]. The advantage of Invisalign aligners not only included tooth movement, but the removable ability of aligners during normal brushing and eating activities has helped improve oral hygiene, as noted by reduction of plaque accumulation, incidences of gingivitis, and periodontitis, which are commonly seen in patients with fixed orthodontic appliances [3,4,5]. As treatment demographics have changed over the years, orthodontic treatment is no longer limited to children and adolescents since more adults seek orthodontic treatment due to its esthetic appeal and everyday practicality [4]. From 1989 to 2012, the number of adult orthodontic patients in the United States and Canada increased by 40%, and a total of 1.4 million patients were recorded in 2014, according to the American Association of Orthodontists. Along with the increasing demand for orthodontic treatment, teeth whitening has also become a major esthetic goal that patients commonly pursue during or closely after treatment concludes [6]. This has influenced current studies to observe the efficacy of dental bleaching agents within the aligners itself.

A common issue patients face with fixed orthodontics is the materials used can easily create various plaque accumulation sites, which subsequently can lead to the formation of potential precursors for white spots, staining, caries, and periodontitis [4]. As the contact of plaque and tooth surface increase in quantity and duration, this interaction can transition into more aggressive biofilms in the gingival pockets, which can lead to inflammation, gingival bleeding, and gingivitis [7]. Past microbiological studies comparing fixed orthodontics to Invisalign technology have found significant plaque decrease with Invisalign treatments due to better compliance to oral hygiene maintenance and ease of access to all tooth surfaces [3,4].

Patients undergoing orthodontic treatments are commonly dissatisfied with their initial tooth shade at the beginning of treatment or may develop teeth discoloration as orthodontic treatments progress. A common contributor of stains on the external surface of the tooth is often poor oral hygiene, smoking, or dietary intake such as coffee or wine [8]. In rare orthodontic cases, the color of the teeth may darken as a result of complications from necrosis or hyperemia of the pulp tissue [9]. As a result, adult orthodontic patients often seek whitening treatment to improve the aesthetic and their overall satisfaction with their smile. Although literature is scarce in relating negative effects in tooth shade to Invisalign treatment, the removable fashion of aligners has presented a greater chance for better oral health, while minimizing the appearance of white sport lesions due to enamel decalcification, a common clinical finding in fixed orthodontic cases [10].

The two most commonly used active ingredients for tooth bleaching are carbamide and hydrogen peroxide, whereby carbamide peroxide (CP) breaks down further into hydrogen peroxide and urea, resulting in the bleaching effects and a rise in the pH of the oral cavity. Urea as a byproduct causes a transient increase in oral pH, which further neutralizes saliva (normal physiologic pH of 6.2–7.6) and contributes to a reduction in plaque formation as well as the incidence of decalcification and caries [11]. The use of CP in both dental practices and at home has been approved by the American Dental Association at a concentration of 10%, which is associated with minimal postoperative sensitivity [12].

Several studies investigated the effects of CP on teeth shade as well as on oral health. Studies only investigating tooth shade change found at least a three-shade improvement after one week of using 10% CP as their instructed bleaching agent [12]. In institutionalized and ambulatory patients with cerebral palsy, applying CP by finger massage for two minutes, three times daily for one month, has resulted in decreased amounts of plaque and calculus deposits. The antibacterial properties of CP were also studied on demineralized fissures injected with lactobacillus as simulants of carious lesions and concluded 10% CP inhibited growth followed by killing the bacteria by penetration of the fissures [12]. Some promising indicators of improved oral health include reduction of gingival bleeding and periodontal probing, which can also be explained by a decrease in plaque accumulation as a result of direct application of 10% CP [13]. Because of its antibacterial effects, the use of CP has expanded to include its application in custom fitted nightguard appliances, which was introduced as a consequence of the increased demand for teeth whitening [14].

Along with the increasing preference for Invisalign, teeth whitening is in greater demand more frequently during or after Invisalign treatment. Based on the current trend of increasing preference of Invisalign adult orthodontic treatments over traditional orthodontic braces and the use of bleaching agents with nightguards, the purpose of this study is to investigate the potential use of 10% CP concurrently with Invisalign trays. The aim of this study is to examine the effects of 10% carbamide peroxide (CP) on tooth shade, plaque index (PI), and gingival index (GI). We hypothesized that the use of 10% CP will significantly enhance tooth shade and improve periodontal health during Invisalign treatments. Our null hypothesis was that tooth bleaching with 10% CP during Invisalign treatment will have no effect on tooth shade or periodontal health indices. To the best of our knowledge, this is the first study that examines the effects of 10% CP application with Invisalign treatments on oral health predictors, such as PI and GI, while assessing changes in tooth shade.

## 2. Materials and Methods

### 2.1. Patient Selection/Recruitment

The study was conducted according to the guidelines of the Declaration of Helsinki and approved by the Institutional Review Board of Western University of Health Sciences (WesternU IRB protocol # FB18/IRB/109, approved on 8 April 2019). Patient recruitment consisted of selection of existing patients at Western University of Health Sciences Dental Center who were actively undergoing Invisalign Treatments. Prior to the study, patient records were reviewed to ensure subjects satisfy the inclusion and exclusion criteria. The inclusion criteria were patients of either sex, 18 years of age or older (with no upper age limits), and who have a minimum of 6 intact teeth in both the mandibular and maxillary arch. Among the 12 required existing teeth, they must include teeth # 7–10 and # 23–26. The exclusion criteria were individuals who had a history of chronic periodontitis (resulting in severe radiographic bone loss) or rampant decay, smoking, currently pregnant, mixed or deciduous dentition, tetracycline-stained teeth, or any anterior restorations (such as composite resins or crowns) on # 7–10 and # 23–26 since ceramics or composite resins will not respond to any bleaching effects.

**Figure 1:**
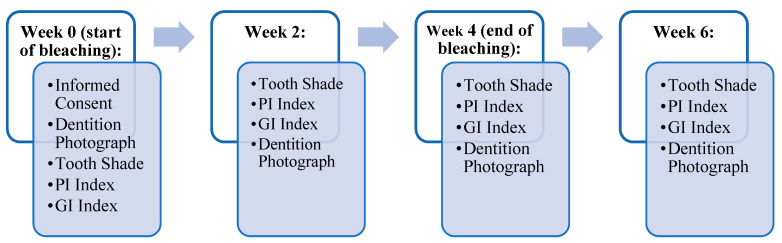
Periodic examinations were conducted bi-weekly to measure the progress of subjects’ oral health.

Total sample size of twenty-eight (n = 28) subjects who underwent Invisalign treatment were randomly divided on the basis of their existing upcoming orthodontic appointments on the clinic schedule into two groups of fourteen (n = 14) subjects each: an experimental group that received the bleaching agent (10% CP) and a control group that did not receive any bleaching agents, and were instructed not to use any over the counter bleaching products as part of their home oral care regimen (Table 1). The experimental group was instructed to apply the bleaching agent, 10% carbamide peroxide (CP) overnight for eight hours daily for four consecutive weeks. Bleaching material application was demonstrated according to the manufacturer’s instructions (Opalescence, Ultradent, UT, USA). Routine oral hygiene care as instructed by their orthodontist were given to all participating subjects at the start of the study and at each biweekly follow-up visit for the duration of this study, which lasted for a total of 6 weeks. Periodic evaluations of shade, plaque index (PI), and gingival index (GI) were performed on all patients at week 0 (baseline) as a pre-operative assessment, at week 2 (midpoint of bleaching), week 4 (end of bleaching), and at week 6 (2 weeks post-bleaching). The teeth of interest for scoring were the upper and lower central and lateral incisors (tooth # 7-# 10 and tooth # 23-# 26). Dentition photographs were taken for case documentation. The overall time scheme of the experiment in terms of assessments of shade, PI, and GI is illustrated in Figure 1. Patients were examined by two calibrated examiners. Kappa coefficient (κ) was used to measure inter-rater agreement.

Informed consent was obtained from all subjects involved in the study. Subjects were informed of common side effects that may arise from the use of teeth whitening agents. Any side effects, such as tooth sensitivity or pain, encountered by subjects were documented in a self-log throughout the study. At each periodic examination, subjects were required to bring their self-logs for observation by the examiners. Subjects were recommended to use Sensodyne ProNamel Toothpaste, containing potassium nitrate and sodium fluoride, to mitigate tooth sensitivity. In the event of an injury were to occur, subjects were advised to contact the principal investigator (PI). After-hours emergency assistance was also provided at the emergency clinic via the dental center answering services.

### 2.2. 10% Carbamide Peroxide Application

The bleaching agent (10% carbamide peroxide) was supplied by Ultradent manufacturer, in 1.2 mL pre-filled Opalescence syringes. Each subject in the experimental group was provided with a two-week supply of bleaching syringes, which were replenished at the two-week examination/follow-up appointment.

### 2.3. Shade Matching Using the VITA Classical A1-D4 Shade Guide

All assessments were determined by calibrated examiners at each visit. Vitapan Classical shade guide was used to assess teeth shade. The shade of the maxillary and mandibular lateral and central incisors was used for consistent assessment (tooth # 7–10 and # 23–26), mainly examining the middle third of the tooth rather than the cervical and incisal third to avoid discrepancies in shade alteration due to any existing gingival recession or incisal wear on the teeth. Shade tabs were labeled one to sixteen (as illustrated in Table 2) and used to shade match all bleached teeth under natural operatory light.

### 2.4. Plaque Index (PI) Using Turesky Modification of the Quigley-Hein Index

The Turesky Modification of the Quigley–Hein Index was used to evaluate the plaque levels on the facial surfaces of both maxillary and mandibular central and lateral incisors that were subjected to the bleaching agent [15]. The scores followed a 0 to five scale using disclosing tablets to visualize plaque accumulation. Plaque scores were recorded by independent examiners at each visit. All scores given by examiners were averaged to determine the index of each bleached tooth at any given point in time.

### 2.5. Gingival Index (GI) Using the Löe and Silness Gingival Index

The Löe and Silness GI was used to assess gingival health by identifying areas of gingival inflammation. The facial cervical/gingival portions of the selected teeth were scored on a scale of zero to three to identify the gingival index per each selected tooth [16]. GI index was recorded by independent examiners at each visit. The scores of gingival indices were averaged to identify the GI for the specified teeth assessed at each given time point.

### 2.6. Statistical Analysis

Data were analyzed using SPSS software 23 (IBM Corp, Armonk, NY, USA), Student-t-test for independent variables, and Mann Whitney U test. Variability within each group was evaluated using descriptive statistics. Differences between and within the groups were analyzed using the appropriate parametric or non-parametric measures as dictated by the results. Shade, plaque, and gingival indices were averaged for each of the two groups at each time point (weeks 0, 2, 4, and 6). Results were reported as a mean ± SD, and *p*-value < 0.05 was considered significant. Two examiners collected the data and calibrated the assessment of such measurements. Cohen’s Kappa coefficient (κ) was used to measure inter-rater agreement.

## 3. Results

### 3.1. Tooth Shade

**Figure 2:**
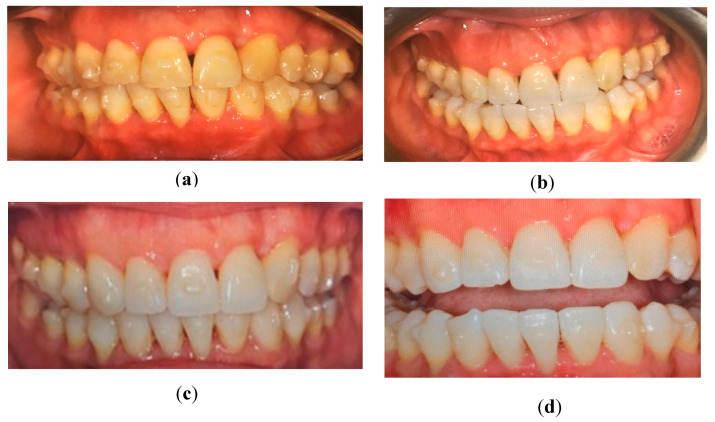
Patient representation at baseline (**a**), after 2 weeks (**b**) of bleaching teeth, after 4 weeks (**c**), and at 6 weeks post-op (**d**).

**Figure 3:**
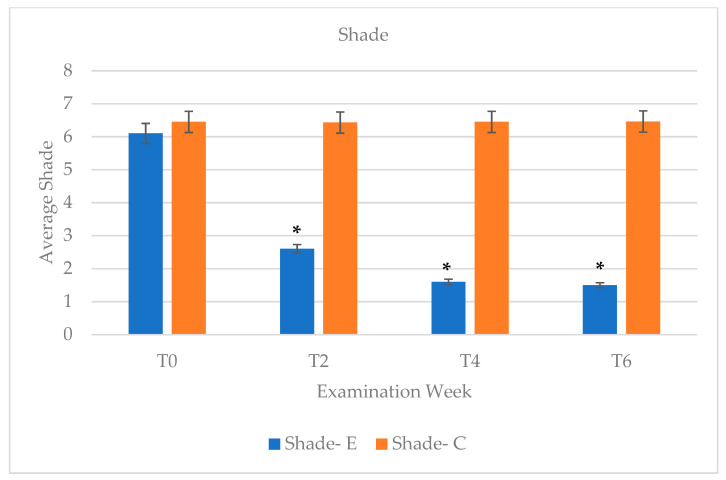
Tooth shade measurements were compared between experimental group (E) and control group (C) and categorized by examination week. Each bar represents an average shade score of each group at each evaluation week. This range of shade measurements received a κ of 0.788 and an inter-exchanger agreement of 87%. (T0—baseline/week 0; T1—week 1; T2—week 2; T4—week 4; T6—week 6) (* *p* < 0.05).

**Figure 4:**
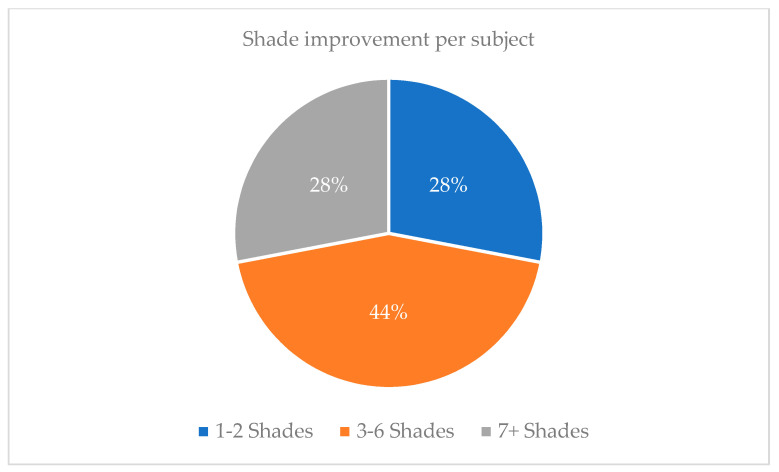
After four weeks of using 10% CP, the subjects experienced variable ranges of shade improvement where 28% improved by 1–2 shades, 44% improved 3–6 shades, and 28% improved by 7+ shades.

The experimental group that had undergone teeth bleaching had an initial shade of 6.1 at baseline time (T0) then showed a significant improvement in shade after week 2 (T2) with a shade number decrease to 2.6 (Figure 2a,b and Figure 3). Evaluations at 2 weeks intervals following showed a slight improvement in shade number to 1.6 at week 4 (T4) and remained constant at 1.5 at week 6 (T6) (Figure 2c,d and Figure 3). At the conclusion of the study, about half of the patients (44%) presented an improvement of 3 to 6 shades, while the remaining subjects experiences improvement of 1 to 2 shades (28%) or 7+ shades (28%) (Figure 4). In contrast, the control group (non-bleaching group) maintained a consistent shade average of 6.43 to 6.46 throughout the duration of the study (Figure 3).

### 3.2. Plaque Index

**Figure 5:**
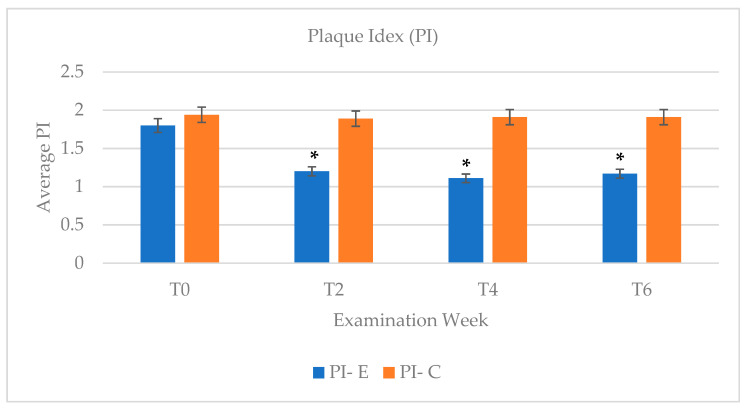
PI measurements were compared between experimental group (E) and control group (C) and categorized by examination week. Each bar represents an average PI score of each group at each evaluation week. The PI measurements received a κ of 0.837 and an inter-examiner agreement of 82%. (T0—baseline/week 0; T1—week 1; T2—week 2; T4—week 4; T6—week 6) (* *p* < 0.05).

The PI was measured periodically as an indicator of oral health improvement. A consistent measurement of 1.89 to 1.94 was identified in the control group for the duration of the study, while there was a significant decrease (*p* < 0.05) in PI in the experimental group at week 2 (T2) compared to the initial baseline measurement of 1.8 (Figure 5). At week 4 (T4) and week 6 (T6), the PI in the experimental group remained consistently low at 1.11 and 1.17, respectively.

### 3.3. Gingival Index (GI)

**Figure 6:**
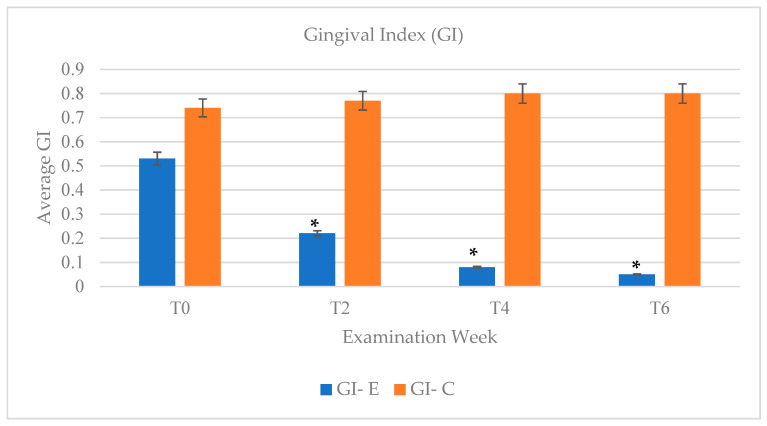
GI measurements were compared between experimental group (E) and control group (C) and categorized by examination week. Each bar represents an average GI score of each group at each evaluation week. The GI measurements received a κ of 0.758 and inter-examiner agreement of 91%. (T0—baseline/week 0; T1—week 1; T2—week 2; T4—week 4; T6—week 6) (* *p* < 0.05).

Gingival index was used as another assessment measure of oral health improvement. The control group maintained a consistent score throughout the study with GI measurements ranging from 0.74 to 0.8 (Figure 6). In contrast, the experimental group showed a significant decrease in GI scores after week two (T2) since results showed a decrease in GI measure of 0.22 in comparison to the initial measurement of 0.53 at T0. GI scores continued to improve in the experimental group at week four (T4) and week six (T6) with GI measurements of 0.08 and 0.05, respectively.

### 3.4. Side Effects

One experimental group subject has reported some minor post-operative sensitivity due to existing gingival recession, which has led to reduction in the amount of time of teeth bleaching per day (<8 h). However, the patient elected to remain in the study and reported minimal or improved sensitivity thereafter.

## 4. Discussion

Several studies have assessed the effectiveness of 10% carbamide peroxide (CP) in improving overall oral gingival health while using the bleaching agent in custom fitted bleaching trays [4,11,15]. In addition, research pertaining to teeth whitening has expanded its potential use and application with nightguards [17]. In response to the increased demand for Invisalign orthodontic treatments and the growing populations’ interest in dental esthetics, studies have continued to expand our knowledge of 10% CP and its multifactorial benefits in orthodontics and general dentistry [5,8,18]. Despite the tight fit provided by Invisalign aligners and the skepticism of using bleaching agent in conjunction with Invisalign trays, it is advantageous to investigate such a technique to provide patients with the benefit of correcting teeth alignment via Invisalign treatments while still improving tooth shade and gingival health [1,2]. Set apart from other studies, this novel pilot study aims to identify the effects of 10% CP use in Invisalign trays on oral periodontal and gingival health measures, such as PI and GI as well as on changes in tooth shade.

In sight of identifying new oral health promoting material, various studies have found CP and other home bleaching systems to be effective in improving tooth shade [13]. Since the use of bleaching agents in soft nightguards was introduced by Haywood and colleagues, take-home whitening systems have been of great use for several years, therefore influencing the methodology of this study. An ongoing debate is the effectiveness of in-office, high-concentrated hydrogen peroxide agents versus low-concentrated agents, which are usually provided in most take-home systems. Due to the vast amount of supporting evidence, low concentration agents are known to be safe without direct professional guidance because of their association with low adverse effects, namely, teeth sensitivity. Our observed results confirmed that the use of 10% concentration of carbamide peroxide only yielded one subject reporting minor teeth sensitivity due to slight preexisting gingival recession. Since there was no reported sensitivity by the remaining experimental subjects, all participants continued to follow the teeth-bleaching instructions in terms of duration of bleaching applications. In addition, key factors to consider with tooth whitening are that shade improvement can be achieved based on consistent concentration of bleaching agent as well as the time lapsed in which the teeth surfaces remain in contact with the bleaching agent [1]. With the duration of bleaching time in consideration, studies that used 10% CP as their designated agent also instructed their participants to use the agent overnight ranging from seven to nine hours over a variable range of consecutive days due to its long reactive half-life [1,4,17,19]. Instructing an overnight wear-time of eight hours was found to be sufficient in producing an average shade improvement of 3.5 among the experimental group just after two weeks of application, followed by one unit of shade improvement at week four (Figure 2 and Figure 3). With an average total of 4.5 shade improvement, this accounts for 44% or participants (Figure 4). Since 78% of participants were reported to display three or more shades of improvement, 10% CP has shown to be effective for tooth whitening overnight (8 h duration) while used in aligner materials.

In addition to the significant shade improvement, CP is frequently compared to other whitening agents, such as hydrogen peroxide based or non-hydrogen peroxide based, in terms of its effects on overall oral health and its chemical degradation in the oral environment [12]. As mentioned earlier, the dissociation of CP produces hydrogen peroxide and urea byproducts, where urea further decomposes into a strong base of CO_2_ ammonia. With the presence of a strong base and strong free radicals, these byproducts are very reactive with organic pigments during the whitening process [19]. With direct application of CP, the release of urea initiates a series of reactions that causes the pH of plaque and saliva to increase to a pH value characteristic to demineralization of enamel and dentine. In effect, this elevation reduces the rate of caries just two hours after application [18]. In addition, hydroxyl radicals are highly reactive with bacterial membrane lipids and DNA, thus initiating bacterial cell death [20]. Thus, by altering plaque microflora, debridement properties of released peroxides are achieved by CP, thereby increasing oxygen availability to promote gingival tissue healing [20].

Following application of 10% CP, reduction in gingival bleeding and gingival inflammation were noted as observed by the improvement in PI and GI indices. In comparison to the results of PI and GI scores in the control groups, which remained consistent (PI = 1.89 to 1.94) throughout the study, the experimental group showed the most significant decrease in PI after 2 weeks post bleaching (Figure 5). At 2 weeks post bleaching, the PI index of the experimental group showed the largest change in PI score with a difference of 0.6 with week two (PI = 1.2) in comparison to an initial PI = 1.8. Similarly gingival health seemed to improve over the 4 weeks-time course with CP bleaching application (Figure 6).

In order to assess any potential relapse in shade, plaque levels, or gingival inflammation, a post-operative examination check at 6 weeks following the initial start of the study, which corresponded to 2 weeks post bleaching, was arranged for all subjects. It has been noted in the literature that most teeth-bleaching cases have satisfactory retention of improved tooth shade without retreatment in at least 43% at 10 years post-treatment [21]. As expected, based on the longevity effects of CP in whitening the teeth, the experimental group retained similar tooth shade at week 6 (Shade = 1.5) compared to the shade attained at week four (Shade = 1.6) (Figure 3). The trend of improvement in PI and GI was overall retained at week six, despite the discontinuation of CP application (Figure 5 and Figure 6), which shows beneficial effects of CP in reducing plaque accumulation and gingival inflammation even 2 weeks post bleaching.

Limitations of this study included a few factors for consideration. Sample size of only 28 subjects in this study (n = 14 in each of the experimental and the control groups), who were concurrently underwent Invisalign treatment and who also qualified to be included in the study based on the specific inclusion and exclusion criteria, was quite small but may serve as a foundational pilot study. In addition, the limited number of available calibrated clinical examiners did not allow for a randomized double blinded type of study. The patients were only randomly assigned by the principal investigator on the basis of their existing upcoming orthodontic appointments until an equal number of participants were reached in each of the experimental and the control group. Throughout the experiment, both the subjects and the examiners knew if teeth bleaching was used or not, which renders the study as non-blinded in nature.

Future direction for research may consider a larger sample size in a double- blinded randomized clinical trials to further investigate the efficacy of carbamide peroxide bleaching during Invisalign treatment. Non-bleaching control group patients may be given placebo gel syringes with no bleaching ingredients to blind subjects about receiving teeth-bleaching agents. Furthermore, while the sample size utilized in this study provided a good representation of the average population with acceptable oral health parameters to undergo teeth whitening by excluding individuals with pre-existing periodontal conditions, those who were smokers, and those who had other systemic conditions that might have predisposed them to periodontal disease, such as diabetes, it might be advantageous for future studies to explore the use of CP in those patients with slightly less than optimal periodontal status. In addition, orthodontic treatment typically uses composite tads for optimal tooth alignment, which requires the Invisalign aligners to be manufactured with wells. Since the bleaching agents work best with direct uniform teeth contact, more studies are needed to identity if the use of bleaching with aligners that adhere directly to teeth provide a better overall outcome in comparison to fabricating custom-fitted bleaching wells within the Invisalign trays. Another area of future research lends itself to explore the possibilities of tooth shade discrepancies using the current approach in this study, especially after the removal of the composite attachments, as there might be slight shade discrepancies between the areas that were previously covered by the composite attachments and the rest of the coronal portion that was exposed to the bleaching agent. If tooth shade discrepancy is to be detected, reapplication of the bleaching agent may be needed at the retention phase of the Invisalign treatment once all the attachments have been removed from the teeth

## 5. Conclusions

The use of 10% carbamide peroxide with Invisalign clear thermoplastic trays seems to be effective in improving tooth shade, plaque, and gingival indices over a 4-weeks period, with no relapse after 2 weeks of discontinuing bleaching. Patient’s compliance with routine oral hygiene care plays an important role in the improvement of periodontal health during and after tooth bleaching.

## Figures and Tables

**Table 1:** Study participants were divided into 2 groups.

Groups	Patient Source	Protocol
Group 1 (Experimental)	Existing Invisalign patients (14 patients)	Invisalign with 10% CP application
Group 2 (control)	Existing Invisalign patients (14 patients)	Invisalign without CP

**Table 2:** Vitapan classical shade guide arranged from lightest to darkest value.

B1	A1	B2	D2	A2	C1	C2	D4	A3	D3	B3	A3.5	B4	C3	A4	C4
1	2	3	4	5	6	7	8	9	10	11	12	13	14	15	16

## Data Availability

The data presented in this study are available on request from the corresponding author.
